# The elimination of trace arsenic via hollow fiber supported liquid membrane: experiment and mathematical model

**DOI:** 10.1038/s41598-021-91326-9

**Published:** 2021-06-03

**Authors:** Sira Suren, Watcharapong Ampronpong, Ura Pancharoen, Kreangkrai Maneeintr

**Affiliations:** 1 Department of Chemical Engineering, Faculty of Engineering, Chulalongkorn University, Bangkok, 10330 Thailand; 2 Department of Mining and Petroleum Engineering, Faculty of Engineering, Chulalongkorn University, Bangkok, 10330 Thailand

**Keywords:** Chemical engineering, Environmental sciences, Mathematics and computing

## Abstract

This work presents the elimination of arsenic ions from synthetic produced water via hollow fiber supported liquid membrane (HFSLM). Results demonstrate that arsenic ions in synthetic wastewater can be successfully treated to meet the wastewater standard as formulated by the Ministry of Industry and the Ministry of Natural Resources and Environment, Thailand. The discharged limit of arsenic from industrial wastewater must not be greater than 250 ppb. In a single-step operation, arsenic ions are extracted and stripped. Percentages of extraction and stripping proved to be 100% and 98%, respectively. Optimum conditions found proved to be 0.68 M Aliquat 336 dissolved in toluene as the liquid membrane, at pH 12 of feed solution, having a mixture of HCl and thiourea as the synergistic strippant, and flow rates of both feed and strippant solutions of 100 mL/min. A mathematical model, developed to predict the final concentration of arsenic ions in feed and strippant solutions, is seen to fit in well with the experimental results.

## Introduction

Produced water from offshore oil and gas production is always contaminated with arsenic, existing in the form of H_3_AsO_3_, H_3_AsO_4_ and H_2_AsO_4_^−^^1,2^. Arsenic is a highly toxic metal causing chronic or acute poisoning in human beings, depending on the quantity received^3^. The amount of arsenic exposed to the environment continuously increases with the quantity of produced water from the offshore oil and gas industry as well as other industries e.g. metallurgical industry and petroleum refining industry^4^. The concentration of arsenic from various wells is found between 1 and 4 ppm (parts per million or mg/L)^5^. Both the Ministry of Industry and the Ministry of Natural Resources and Environment, Thailand, have stipulated that the amount of arsenic in wastewater discharges should be no higher than 250 ppb^6^.

In practice, conventional methods i.e. adsorption, ion exchange, coagulation and precipitation have been introduced to treat arsenic and other toxic metals in the produced water. However, sometimes the concentration of toxic metals in produced water after treatment is still over the wastewater discharged standard^7^. Inbaraj et al.^8^ recorded that conventional methods are always ineffective when concentration of the target metal ions is very low, at ppb level. In effect, treated produced water, from offshore oil and gas production, which has toxic metal over the discharge limits is re-injected into the rock formations from where the oil and gas originated^7^. Nevertheless, reinjecting produced water into rock formations is still risky since toxic metals can spill into the environment. An alternative method is therefore required.

In recent years, new adsorbents for removing toxic metals have been developed^9,10^. Magnetic MCM-41 nanosorbents, for instance, have been used to selectively remove chromium(VI) oxyanions from various aqueous systems^9^. However, the concentration of chromium(VI) oxyanions that remained in the aqueous solutions was still over the discharged limit required.

As for the elimination of target ions from various solutions, at extremely low concentration of ppb levels, the HFSLM system is most recommended. Guell et al.^11^ applied HFSLM for the removal and preconcentration of chromium(VI) from synthetic wastewater using tri-octyl methyl ammonium chloride (Aliquat 336) dissolved in a mixture of dodecane and 4% dodecanol as the liquid membrane and HNO_3_ as the strippant. Results showed that HFSLM could remove chromium(VI) from industrial waters and spiked natural waters at an extremely low concentration of µg/L level. The system enabled both the separation and enrichment of the metal within a single-step operation. Ni’am et al.^12^ utilized HFSLM for the extraction and stripping of dysprosium, praseodymium and neodymium from waste magnets in a single-step operation. Percentages of extraction of dysprosium, praseodymium and neodymium reached 99%, 86%, and 59%, respectively. Meanwhile, the percentages of stripping of dysprosium, praseodymium and neodymium reached 15%, 56%, and 63%, respectively. Scott et al.^13^ used HFSLM applying Aliquat 336 dissolved in 3% dodecanol/dodecane as the liquid membrane for extraction of radioisotope ^48^V from aqueous solutions spiked with chemically similar species. HFSLM proved to be effective in extracting ^48^V (71%) at the concentration of ppt level.

Other advantages of HFSLM over traditional methods involve high selectivity^14,15^, less consumption of extractant and solvent used, low energy consumption as well as low capital and operating costs^16^. The high surface area of HFSLM provides high mass transfer rate of elimination^17,18^. HFSLM can be applied in many industries such as wastewater treatment^11,19^, food and biological processing^17,20^, and pharmaceutical^14^. Rathore et al.^19^ introduced polypropylene HFSLM to eliminate and recover plutonium from aqueous acidic wastes generated in nuclear chemical facilities. The extractant n-butyle phosphate (TBP) is dissolved in dodecane and used as the liquid membrane. In the presence of various fission products (Cs-137, Ru-106 and Eu-154), the selective permeation of plutonium(V) into the strippant phase comprised of 0.1 M NH_2_OH·HCl in 0.3 M HNO_3_ proved to be greater than 90%. Romero-González et al.^21^ applied HFSLM to simultaneously analyze pesticide residues in vegetables e.g. cucumber, tomato and pepper by liquid chromatography (LC) coupled with electrospray mass spectrometry (MS). The study showed that the HFSLM technique required minimal sample preparation and solvent consumption. Detection and quantification limits, in the range of 0.06 to 2.7 µg/kg and 0.2 to 9.0 µg/kg, have been found low enough to determine pesticide residues at concentrations $\le$ the maximum residue levels (MRLs) specified by the European Union. Sunsandee et al.^14^ utilized HFSLM to selectively separate enantiomer (S)-amlodipine from pharmaceutical wastewater, thus achieving percentages of extraction and stripping of 82.0 and 76.0%, respectively.

In the application of HFSLM, connecting the hollow fiber modules in series or in parallel can increase the capacity of elimination^22^. As per the several studies mentioned above, it is acknowledged that after other traditional methods have been carried out, HFSLM is an effective method for use as a secondary process to fulfill their drawbacks.

With regard to the efficient elimination of metal ions, HFSLM strongly depends on the types of extractants and strippants used as well as the acidity of feed solution. Hence, these parameters have been reviewed and applied in this work. Pancharoen et al., for instance, highlighted the extraction (about 90%) and stripping (about 70%) of arsenic ions using Aliquat 336 as the extractant and NaOH as the strippant^2^. Jantunen et al.^23^ separated arsenic from concentrated sulfuric acid using TBP as the extractant attaining 83.7% of arsenic extracted in three-stages of separation. As for the stripping of various metal ions, thiourea has been found to be effective^24–26^. Fábrega and Mansur^27^ found that thiourea could strip almost 100 percent of mercury(II) from mercury-Aliquat 336 complex.

HFSLM has attracted the interest of many researchers in its application for the treatment of polluted wastewater. For application on an industrial scale, however, solid mathematical models are required. Such models help to provide an essential guideline for understanding the transport mechanism of target ions across the liquid membrane. Such models can also help to predict separation time and promote efficiency. Mathematical models are vital tools for estimating the cost and scaling up the process of separation.

Several mathematical models have been developed to explain the transport of metal ions across HFSLM. Yet, only a few models have been generated to describe the transport of target ions on both feed and strippant sides. Jagdale et al.^28^, for example, developed a mathematical model for predicting the extraction and stripping of neodymium(III) via HFSLM. The model was implemented by considering mass accumulation, convection and diffusion but chemical reaction was ignored. In addition, our previous mathematical model, which evolved in order to predict the extraction and stripping of mercury(II) as well as lead(II) via HFSLM, was based on chemical reactions, mass accumulation and mass convection, though diffusion was ignored^29^. The above-mentioned models ignored either the chemical reaction or diffusion. Subsequently, the models could not predict the extraction and stripping of some target ions, which have different chemical and physical properties. Examples of mathematical models for predicting the elimination of target ions via HFSLM are summarized in Table 1.

**Table 1:** Mathematical models used for predicting the elimination of metal ions via HFSLM.

Authors	Target ions	Extractants/strippants	Included parameters				Model predicted/%deviation
			CT	DT	MA	R	
Kandwal et al.^30^	Cs(I)	CNC/distilled water	✓	✓	–	–	Extraction/n/a
Vernekar et al.^31^	Co(II)	D2EHPA/H_2_SO_4_	✓	✓	–	–	Extraction/n/a
Choi et al.^32^	Co(II) and Ni(II)	HEH/H_2_SO_4_	✓	✓	✓	–	Extraction/n/a
Yang and Kocherginsky^33^	Cu(II)	LIX54/H_2_SO_4_	✓	✓	–	✓	Extraction/n/a
Jagdale et al.^28^	Nd(III)	TODGA/distillated water	✓	✓	✓	–	Extraction and stripping/n/a
Suren et al.^29^	Hg(II) and Pb(II)	Aliquat 336 for Hg(II), D2EHPA for Pb(II)/thiourea for Hg(II), HCl for Pb(II)	✓	–	✓	✓	Extraction/2%, and stripping 5%
This work	As(V)	Aliquat 336/HCl	✓	✓	✓	✓	Extraction/2%, and stripping 4%

CT, convection transport; DT, diffusion transport; MA, mass accumulation; R, reaction.

This work investigates the elimination of a very low concentration of arsenic ions from synthetic produced water via HFSLM. The parameters studied are as follows: types and concentration of extractants used to prepare the liquid membrane, pH of feed solution, types and concentration of the strippant solutions, and flow rates of feed and strippant solutions. Respond surface has also been applied in order to maximize the efficiency of treating arsenic as well as minimize the cost of chemicals. Moreover, a mathematical model, based on conservation of mass considering axial convection, diffusion, reactions at the liquid–membrane interfaces, and mass accumulation, has been developed to predict the extraction and stripping of arsenic ions.

## Modeling: transport of arsenic ions

### Transport mechanisms of arsenic ions across HFSLM

**Figure 1:**
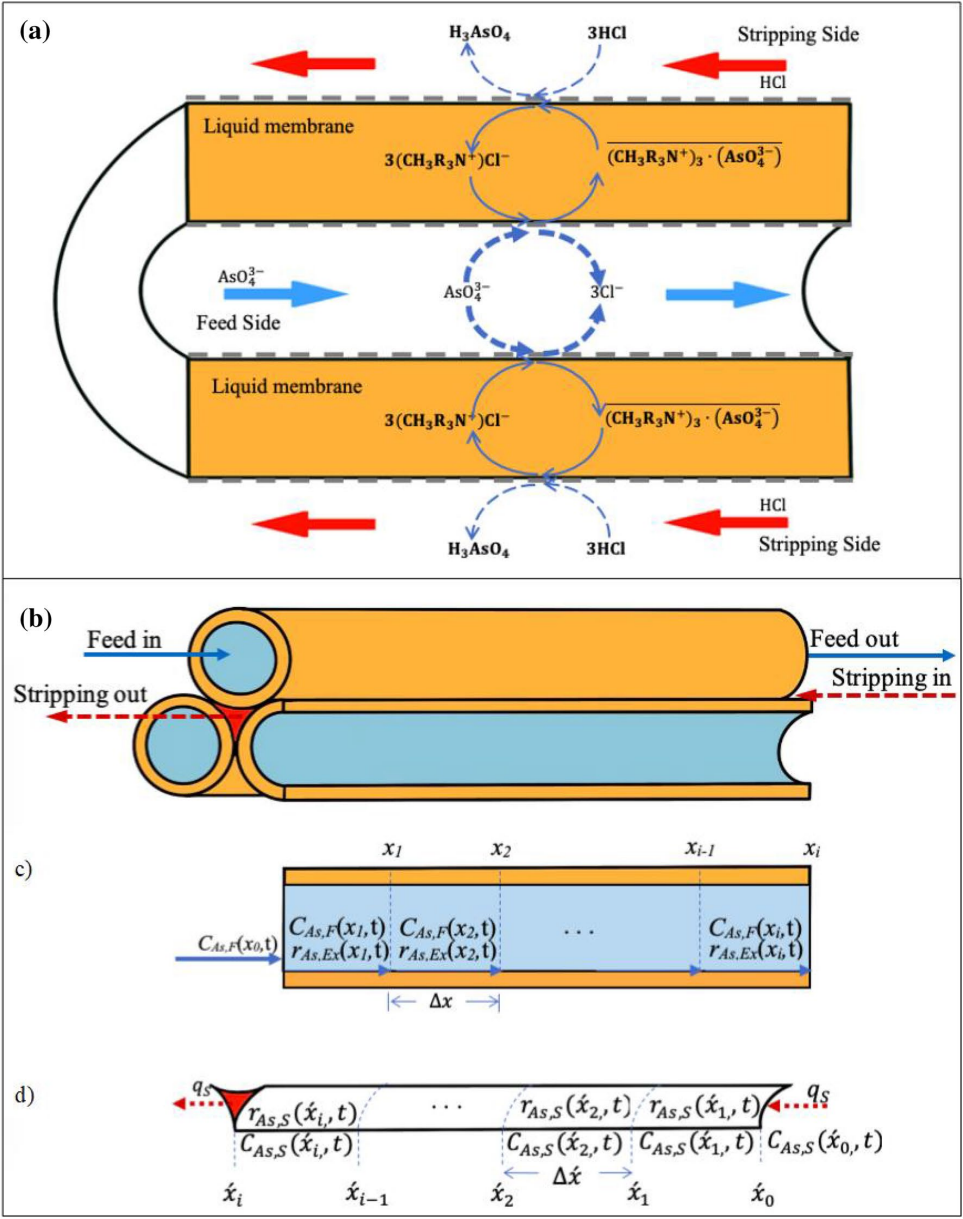
Schema of arsenic ions transport across HFSLM: (**a**) mechanisms of extraction and stripping. (**b**) Tube and shell sides of the hollow fibers. (**c**) Tube side. (**d**) Shell side.

Arsenic in co-produced water exits in various forms, depending on the pH. At pH lower than 2, arsenic(III) exits in its undissociated forms such as H_3_AsO_4_. Arsenic(V) predominantly exists as anions H_2_AsO_4_^−^ at pH between 3 and 6, and exists as ${\mathrm{AsO}}_{4}^{3-}$ at pH between 12 and 14^34^. In Fig. 1a, a schematic diagram of arsenic ions transport across HFSLM is shown. The HFSLM system is comprised of a feed phase (an aqueous solution containing arsenic ions), a strippant phase, and a supported liquid membrane phase embedded with an organic extractant, which separates both feed and strippant phases. As shown in Fig. 1a, ${\mathrm{AsO}}_{4}^{3-}$ reacts with CH_3_R_3_N^+^Cl^−^ (Aliquat 336) at the feed–liquid–membrane interface forming an arsenic-extractant complex species $\overline{{\left({\mathrm{CH}}_{3}{\mathrm{R}}_{3}{\mathrm{N}}^{+}\right)}_{3}\cdot \left({\mathrm{AsO}}_{4}^{3-}\right)}$. Then, $\overline{{\left({\mathrm{CH}}_{3}{\mathrm{R}}_{3}{\mathrm{N}}^{+}\right)}_{3}\cdot \left({\mathrm{AsO}}_{4}^{3-}\right)}$ diffuses across the liquid membrane to the liquid–membrane–strippant interface to react with the strippant HCl. Subsequently, arsenic ions are stripped into the strippant phase. Thus, arsenic ions can be simultaneously extracted and stripped in a single-step operation. The rate of transport of arsenic ions is governed by the concentration gradient between feed and strippant phases.

The extraction reaction of ${\mathrm{AsO}}_{4}^{3-}$ and CH_3_R_3_N^+^Cl^–^ can be expressed as in Eq. (1):

1 $${\mathrm{AsO}}_{4}^{3-}+3\left({\mathrm{CH}}_{3}{\mathrm{R}}_{3}{\mathrm{N}}^{+}\right){\mathrm{Cl}}^{-}\rightleftharpoons \overline{{\left({\mathrm{CH}}_{3}{\mathrm{R}}_{3}{\mathrm{N}}^{+}\right)}_{3}\cdot \left({\mathrm{AsO}}_{4}^{3-}\right)}+{3\mathrm{Cl}}^{-}$$

The stripping reaction of the arsenic-extractant complex and HCl can be expressed as in Eq. (2):

2 $$\overline{{\left({\mathrm{CH}}_{3}{\mathrm{R}}_{3}{\mathrm{N}}^{+}\right)}_{3}\cdot \left({\mathrm{AsO}}_{4}^{3-}\right)}+3\mathrm{HCl}\rightleftharpoons 3\left({\mathrm{CH}}_{3}{\mathrm{R}}_{3}{\mathrm{N}}^{+}\right){\mathrm{Cl}}^{-}+{\mathrm{H}}_{3}{\mathrm{AsO}}_{4}$$

The reaction rate of extraction of arsenic ions (*r*_*As,Ex*_) can be expressed as:

3 $${r}_{As,Ex}\left(x,t\right)={-k}_{Ex}{C}_{As,F}^{m}\left(x,t\right)$$

where *x* is the longitudinal axis of the hollow fiber in the feed phase, *k*_*Ex*_ is the reaction rate constant of extraction, *t* is the elimination time, *C*_*As,F*_ is the concentration of arsenic ions in the feed phase (mg/L) and *m* is the reaction order of extraction.

In the strippant phase, the strippant is kept at excess concentration. Hence, the total reaction of arsenic stripping in Eq. (2) can be considered as forward reaction. Thus, the reaction rate of arsenic stripping (*r*_*As,S*_) becomes:

4 $${r}_{As,S}\left(\acute{x},t\right)={k}_{S}{C}_{As,S}^{n}\left(\acute{x},t\right)$$

where $\acute{x}$ is the longitudinal axis of the hollow fiber in the strippant phase, *k*_*S*_ is the reaction rate constant of stripping, *C*_*As,S*_ is the concentration of arsenic ions in the strippant phase (mg/L) and *n* is the reaction order of stripping.

In some cases, the elimination of target ions via liquid membrane can be enhanced by using a mixture of substances to induce a synergistic effect. To determine whether elimination using a mixture of substances is synergistic elimination or not, the synergistic coefficient *R,* in terms of distribution ratio, is defined as shown in Eq. (5)^35^. The greater the synergistic coefficient of the synergistic system is, the greater the elimination is.

5 $$R=\frac{{D}_{max}}{{D}_{A}+{D}_{B}}$$

where *D*_*max*_ refers to the maximum distribution ratio of the synergistic system (using a mixture of the substances A and B) to eliminate the specified ions. *D*_*A*_ stands for the distribution ratio of elimination using the single substance A, and *D*_*B*_ is the distribution ratio of elimination using the single substance B. If the synergistic coefficient *R* > 1, synergistic elimination is confirmed.

### Development of the mathematical model

A mathematical model for predicting the extraction and stripping of arsenic ions across HFSLM is generated from the conservation of mass at tiny segments of the hollow fibers, as shown in Fig. 1b–d. Parameters in the model include axial convection, diffusion, reactions at the liquid–membrane interfaces, and mass accumulation.

The mathematical model for extraction on the feed side is generated based on the following hypotheses:

1. Temperature, pressure and volume of the feed phase, inside the tube, are constant.
2. The inside radius of the hollow-fiber is tiny. Therefore, the concentration profile of arsenic ions in the radial direction is constant, meaning that the diffusion fluxes of the ions in the feed phase occur only in the axial direction.
3. The extractant in the liquid membrane phase is kept at excess concentration and arsenic ions are transferred into the strippant solution continuously. Therefore, extraction reaction can be considered as forward reaction.
4. Extraction reaction takes place at the feed–liquid–membrane interface along the length of the hollow fiber.
5. Only the arsenic-extractant complex, which is formed due to the extraction reaction, not arsenic ions, can transport into the liquid membrane phase.

In the case of a mathematical model for the stripping of arsenic ions into the strippant phase, a model is generated based on the hypotheses as follows:

1. Temperature, pressure and volume of the strippant phase, in the shell, are constant.
2. Stripping reaction occurs at the liquid–membrane–strippant interface along the length of the hollow fiber.
3. Only arsenic ions according to the stripping reaction at the interface, not the arsenic-extractant complex, can be stripped into the strippant solution.
4. The strippant is kept at excess concentration. Therefore, stripping reaction can be considered as forward reaction.

In the feed phase, the conservation of mass for arsenic ions in each tiny segment (Δ*x*), see Fig. 1c, is defined as Eqs. (6) and (7)^36^:

6 $$\left[\mathrm{Rate\, of \,mass \,transport \,into \,the \,system \,by \,convection}\right]- \left[\mathrm{Rate \,of \,mass \,transport \,out \,of \,the \,system \,by \,convection}\right]+ \left[\mathrm{Rate \,of \,mass \,transport \,through \,the \,system \,by \,diffusion}\right]- [\mathrm{Rate \,of \,mass \,extracted \,by \,extraction \,reaction}] = [\mathrm{Rate \,of \,mass \,accumulation \,within \,the \,system}]$$

7 $${q}_{F}{C}_{As,F}\left({x}_{i-1},t\right)-{q}_{F}{C}_{As,F}\left({x}_{i},t\right)+\frac{{A}_{c,F}{\mathfrak{D}}_{As,F}}{\Delta x}\left({C}_{As,F}\left({x}_{i},t\right)-{C}_{As,F}\left({x}_{i-1},t\right)\right)+{r}_{As,Ex}\left({x}_{i},t\right){V}_{F}={V}_{F}\frac{d{C}_{As,F}\left({x}_{i},t\right)}{dt}$$

where *q*_*F*_ is the volumetric flow rate of the feed solution, *i* is the number of tiny segments divided, *A*_*c,F*_ is the cross-sectional area of the tube, *V*_*F*_ is the volume of a tiny segment of the hollow fiber in the tube side, and ${\mathfrak{D}}_{As,F}$ is the mass diffusivity of arsenic ions in the feed solution, which can be estimated by Eq. (8)^37^:

8 $${\mathfrak{D}}_{As}=\frac{7.4\times {10}^{-8}{\left(\varnothing M\right)}^{1/2}T}{\eta {V}_{A}^{0.6}}$$

where $\mathrm{\varnothing }$ is the solvent association factor and is equal to 2.6, *M* is the solvent molecular weight (g/mol), *T* is the temperature (K), *η* is the dynamic viscosity of the solvent (cP), and *V*_*A*_ is the molar volume of solute A at its boiling temperature (cm^3^/mol).

Linearizing reaction rate in Eq. (3), as described in Appendix A in the Supplementary Information online, and then substituting in Eq. (7) yields:

9 $$\frac{{V}_{F}}{{q}_{F}}\frac{d{C}_{As,F}\left({x}_{i},t\right)}{dt}=\left(1-\frac{{A}_{c,F}{\mathfrak{D}}_{As,F}}{\Delta x}\right){C}_{As,F}\left({x}_{i-1},t\right)-\left(1+\frac{{V}_{F}\Omega }{{q}_{F}}-\frac{{A}_{c,F}{\mathfrak{D}}_{As,F}}{\Delta x}\right){C}_{As,F}\left({x}_{i},t\right)-\frac{{V}_{F}\psi }{{q}_{F}}$$

where $$\Omega =m{k}_{Ex}{C}_{As,F}^{m-1}(\mathrm{0,0}), \psi =\left(1-m\right){k}_{Ex}{C}_{As,F}^{m}(\mathrm{0,0}).$$

The conservation of mass of arsenic ions in the tiny segments 1, 2, 3, …, *i* in the feed side based on Eq. (9) is as follows:

10 $$\frac{{V}_{F}}{{q}_{F}}\frac{d{C}_{As,F}\left({x}_{1},t\right)}{dt}=\left(1-\frac{{A}_{c,F}{\mathfrak{D}}_{As,F}}{\Delta x}\right){C}_{As,F}\left({x}_{0},t\right)-\left(1+\frac{{V}_{F}\Omega }{{q}_{F}}-\frac{{A}_{c,F}{\mathfrak{D}}_{As,F}}{\Delta x}\right){C}_{As,F}\left({x}_{1},t\right)-\frac{{V}_{F}\psi }{{q}_{F}}$$

11 $$\frac{{V}_{F}}{{q}_{F}}\frac{d{C}_{As,F}\left({x}_{2},t\right)}{dt}=\left(1-\frac{{A}_{c,F}{\mathfrak{D}}_{As,F}}{\Delta x}\right){C}_{As,F}\left({x}_{1},t\right)-\left(1+\frac{{V}_{F}\Omega }{{q}_{F}}-\frac{{A}_{c,F}{\mathfrak{D}}_{As,F}}{\Delta x}\right){C}_{As,F}\left({x}_{2},t\right)-\frac{{V}_{F}\psi }{{q}_{F}}$$

12 $$\frac{{V}_{F}}{{q}_{F}}\frac{d{C}_{As,F}\left({x}_{3},t\right)}{dt}=\left(1-\frac{{A}_{c,F}{\mathfrak{D}}_{As,F}}{\Delta x}\right){C}_{As,F}\left({x}_{3},t\right)-\left(1+\frac{{V}_{F}\Omega }{{q}_{F}}-\frac{{A}_{c,F}{\mathfrak{D}}_{As,F}}{\Delta x}\right){C}_{As,F}\left({x}_{3},t\right)-\frac{{V}_{F}\psi }{{q}_{F}}$$

13 $$\frac{{V}_{F}}{{q}_{F}}\frac{d{C}_{As,F}\left({x}_{i},t\right)}{dt}=\left(1-\frac{{A}_{c,F}{\mathfrak{D}}_{As,F}}{\Delta x}\right){C}_{As,F}\left({x}_{i-1},t\right)-\left(1+\frac{{V}_{F}\Omega }{{q}_{F}}-\frac{{A}_{c,F}{\mathfrak{D}}_{As,F}}{\Delta x}\right){C}_{As,F}\left({x}_{i},t\right)-\frac{{V}_{F}\psi }{{q}_{F}}$$

Solving the series of differential equations i.e. Eqs. (10) to (13) using the concept of the Generating Function, as explained in Appendix A in the Supplementary Information online, obtains Eq. (14), which is used to predict the concentration of arsenic ions in the outlet feed solution, C_*As,F*_(*x*_*i*_*,t*).

14 $${\mathcal{C}}_{As,F}\left({x}_{i},t\right)={-\mathcal{C}}_{As,F}\left(\mathrm{0,0}\right){e}^{-\vartheta \frac{t{q}_{F}}{{V}_{F}}}\sum_{k=1}^{i}\frac{1}{\left(i-k\right)!}{\left(\frac{1}{\vartheta }\right)}^{k}{\left(\frac{t{q}_{F}}{{V}_{F}}\right)}^{i-k}{\omega }^{i}+\frac{{V}_{F}\psi {e}^{-\vartheta \frac{t{q}_{F}}{{V}_{F}}}}{{q}_{F}\vartheta }\sum_{k=1}^{i}\frac{1}{\left(i-k\right)!}{\left(\frac{t{q}_{F}\omega }{{V}_{F}}\right)}^{i-k}\sum_{l=1}^{k}{\left(\frac{\omega }{\vartheta }\right)}^{l-1}-\frac{{V}_{F}\psi \sum_{k=1}^{i}{\omega }^{i-k}}{{q}_{F}{\vartheta }^{i-k+1}}+{\mathcal{C}}_{As,F}\left(\mathrm{0,0}\right){\left(\frac{\omega }{\vartheta }\right)}^{i}$$

where $$\omega =1-\frac{{A}_{c,F}{\mathfrak{D}}_{As,F}}{\Delta x}, \vartheta =1+\frac{{V}_{F}\Omega }{{q}_{F}}-\frac{{A}_{c,F}{\mathfrak{D}}_{As,F}}{\Delta x}.$$

*C*_*As,F*_ (0,0) stands for the concentration of arsenic ions in the inlet feed solution.

In the strippant phase, based on Eq. (6), the conservation of mass of arsenic ions in the tiny segment (Δ$\acute{x}$), see Fig. 1d, is written as:

15 $${q}_{S}{C}_{As,S}\left({\acute{x}}_{i-1},t\right)-{q}_{S}{C}_{As,S}\left({\acute{x}}_{i},t\right)+\frac{{A}_{c,S}{\mathfrak{D}}_{As,S}}{{\acute{\Delta x}}_{i}}\left({C}_{As,S}\left({\acute{x}}_{i},t\right)-{C}_{As,S}\left({\acute{x}}_{i-1},t\right)\right)+{r}_{As,S}\left({\acute{x}}_{i},t\right){V}_{S}={V}_{S}\frac{d{C}_{As,S}\left({\acute{x}}_{i},t\right)}{dt}$$

where *q*_*S*_ is the volumetric flow rate of the strippant solution, *A*_*c,S*_ is the cross-sectional area of the shell side of the hollow fiber, *V*_*S*_ is the volume of a tiny segment of the shell side of the hollow fiber, and ${\mathfrak{D}}_{As,S}$ is the mass diffusivity of arsenic ions in the strippant solution, which is calculated using Eq. (8).

Linearizing the reaction rate of stripping (*r*_*As,S*_) in Eq. (4), using the Taylor series as described in Appendix A in the Supplementary Information online, and merging with Eq. (15) achieves:

16 $$\frac{{V}_{S}}{{q}_{S}}\frac{d{C}_{As,S}\left({\acute{x}}_{i},t\right)}{dt}=\left(1-\frac{{A}_{c,S}{\mathfrak{D}}_{As,S}}{\Delta x}\right){C}_{As,S}\left({\acute{x}}_{i-1},t\right)-\left(1-\frac{{V}_{S}\sigma }{{q}_{S}}-\frac{{A}_{c,S}{\mathfrak{D}}_{As,S}}{\acute{\Delta x}}\right){C}_{As,S}\left({\acute{x}}_{i},t\right)+\frac{{V}_{S}\delta }{{q}_{S}}$$

where $$\sigma =n{k}_{S}{C}_{As,S}^{n-1}(\mathrm{0,0})$$, and $$\delta =\left(1-n\right){k}_{S}{C}_{As,S}^{m}(\mathrm{0,0})$$.

The conservation of mass of arsenic ions in the tiny segments 1, 2, 3, …, *i* based on Eq. (16) is as follows:

17 $$\frac{{V}_{S}}{{q}_{S}}\frac{d{C}_{As,S}\left({\acute{x}}_{1},t\right)}{dt}=\left(1-\frac{{A}_{c,S}{\mathfrak{D}}_{As,S}}{\Delta \acute{x}}\right){C}_{As,S}\left({\acute{x}}_{0},t\right)-\left(1-\frac{{V}_{S}\sigma }{{q}_{S}}-\frac{{A}_{c,S}{\mathfrak{D}}_{As,S}}{\Delta \acute{x}}\right){C}_{As,S}\left({\acute{x}}_{1},t\right)+\frac{{V}_{S}\delta }{{q}_{S}}$$

18 $$\frac{{V}_{S}}{{q}_{S}}\frac{d{C}_{As,S}\left({\acute{x}}_{2},t\right)}{dt}=\left(1-\frac{{A}_{c,S}{\mathfrak{D}}_{As,S}}{\acute{\Delta x}}\right){C}_{As,S}\left({\acute{x}}_{1},t\right)-\left(1-\frac{{V}_{S}\sigma }{{q}_{S}}-\frac{{A}_{c,S}{\mathfrak{D}}_{As,S}}{\acute{\Delta x}}\right){C}_{As,S}\left({\acute{x}}_{2},t\right)+\frac{{V}_{S}\delta }{{q}_{S}}$$

19 $$\frac{{V}_{S}}{{q}_{S}}\frac{d{C}_{As,S}\left({\acute{x}}_{3},t\right)}{dt}=\left(1-\frac{{A}_{c,S}{\mathfrak{D}}_{As,S}}{\acute{\Delta x}}\right){C}_{As,S}\left({\acute{x}}_{2},t\right)-\left(1-\frac{{V}_{S}\sigma }{{q}_{S}}-\frac{{A}_{c,S}{\mathfrak{D}}_{As,S}}{\acute{\Delta x}}\right){C}_{As,S}\left({\acute{x}}_{3},t\right)+\frac{{V}_{S}\delta }{{q}_{S}}$$

20 $$\frac{{V}_{S}}{{q}_{S}}\frac{d{C}_{As,S}\left({\acute{x}}_{i},t\right)}{dt}=\left(1-\frac{{A}_{c,S}{\mathfrak{D}}_{As,S}}{\Delta x}\right){C}_{As,S}\left({\acute{x}}_{i},t\right)-\left(1-\frac{{V}_{S}\sigma }{{q}_{St}}-\frac{{A}_{c,S}{\mathfrak{D}}_{As,S}}{\acute{\Delta x}}\right){C}_{As,S}\left({\acute{x}}_{i},t\right)+\frac{{V}_{S}\delta }{{q}_{S}}$$

Solving the series of differential equations i.e. Equations (17) to (20), using the concept of Generating Function, as explained in Appendix A in the Supplementary Information online, yields Eq. (21), which is used to determine the concentration of arsenic ions in the outlet strippant solution, *C*_*As,S*_ (*x′,t*).

21 $${C}_{As,S}\left({\acute{x}}_{i},t\right)={-\mathcal{C}}_{As,S}\left(\mathrm{0,0}\right){e}^{-\zeta \frac{t{Q}_{S}}{{V}_{S}}}\sum_{k=1}^{i}\frac{1}{\left(i-k\right)!}{\left(\frac{1}{\zeta }\right)}^{k}\cdot {\left(\frac{t{Q}_{S}}{{V}_{S}}\right)}^{i-k}{\varpi }^{i}-\frac{\delta {V}_{S}{e}^{-\zeta \frac{{V}_{S}}{{Q}_{S}}}}{{Q}_{S}\lambda }\sum_{k=1}^{i}\frac{1}{\left(i-k\right)}{\left(\frac{t{Q}_{S}\varpi }{{V}_{S}}\right)}^{i-k}\sum_{l=1}^{k}{\left(\frac{\varpi }{\zeta }\right)}^{l-1}+\frac{\delta {V}_{S}}{{Q}_{S}}\sum_{k=1}^{i}\frac{{\varpi }^{i-k}}{{\zeta }^{i-k+1}}+{C}_{As,S}\left(\mathrm{0,0}\right){\left(\frac{\varpi }{\zeta }\right)}^{i}$$

where $$\varpi =1-\frac{{A}_{c,S}{\mathfrak{D}}_{As,S}}{\Delta \acute{x}}, \zeta =\left(1-\frac{{V}_{S}\sigma }{{q}_{S}}-\frac{{A}_{c,S}{\mathfrak{D}}_{As,S}}{\Delta \acute{x}}\right).$$

*C*_*As,S*_ (0,0) is the concentration of arsenic ions in the strippant phase at time zero, which can be calculated by Eq. (A.23)^36^, see Appendix A in the Supplementary Information online.

The validity of the mathematical model is verified by the experimental results, and the percent average relative deviation (%ARD) is as shown in Eq. (22):

22 $$\%ARD=\frac{1}{N}\sum_{i=1}^{N}\left|\frac{{C}_{Expt.}-{C}_{Model.}}{{C}_{Expt.}}\right|\times 100$$

where *N* is the number of experimental data, *C*_*Expt.*_ and *C*_*Model*_ represent the concentration of arsenic ions obtained from the experiment and the mathematical model, respectively.

## Experimental

### Chemicals

H_3_AsO_4_ was used to synthesize the produced water as feed solution. The initial concentration of H_3_AsO_4_ in the inlet feed solution was 4000 ppb. Both NaOH and HCl were used to adjust the pH of the feed solution and to study the types of strippant solutions. Three types of strippant solutions were investigated: NaOH, HCl and thiourea. Types of extractants studied included Aliquat 336, TBP, DEHP (Bis(2-ethylhexyl) phthalate), Cyanex 471 (tri-isobutylphosphine sulphide) and TOPO (tri-n-octylphosphine oxide). Toluene, as the diluent, was used to dissolve the extractant. All reagents are of AR grade.

### Apparatus

**Figure 2:**
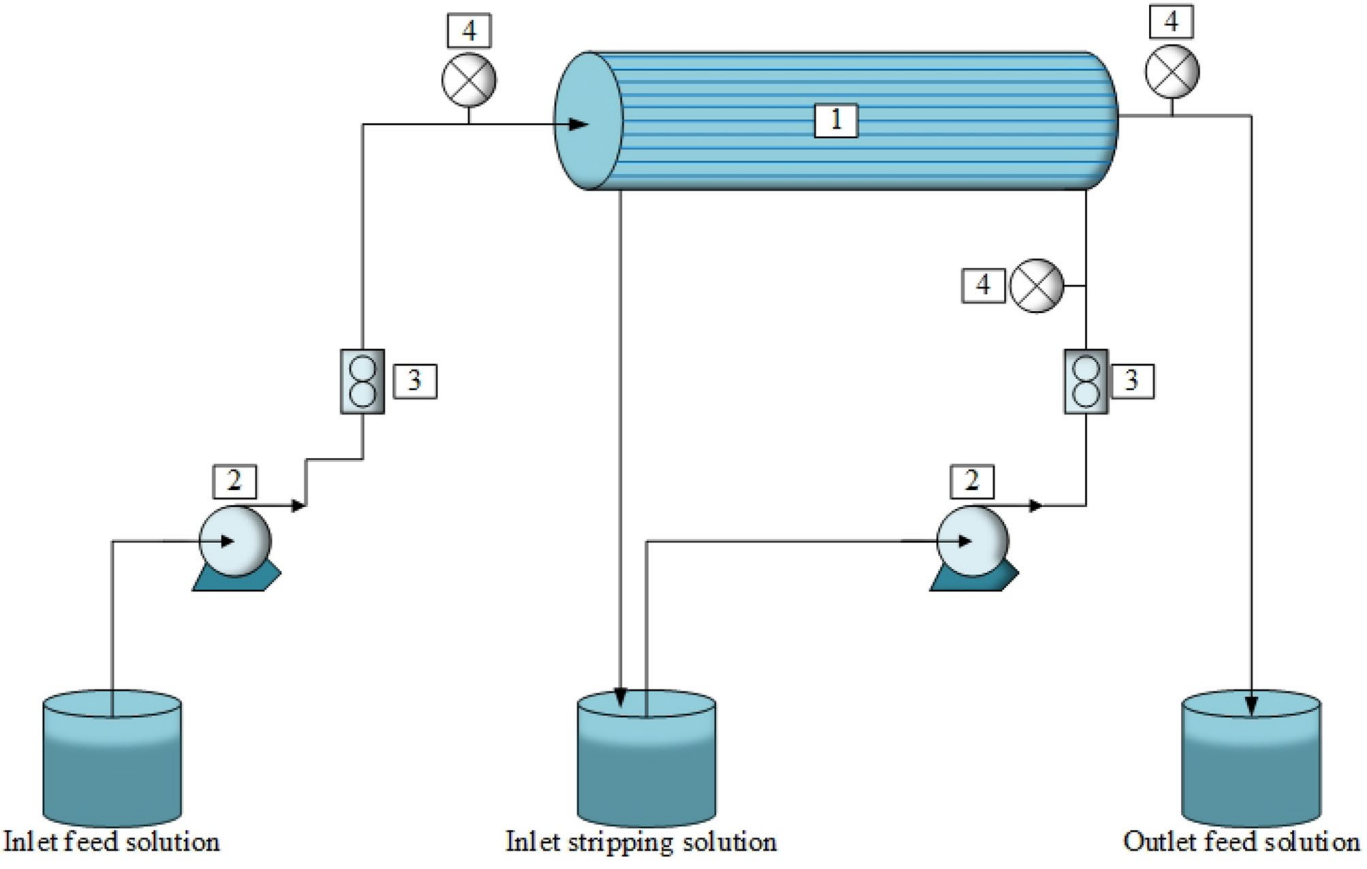
Schema of elimination of arsenic ions via HFSLM: (1) hollow fiber module. (2) Gear pumps. (3) Flow meters. (4) Pressure gauges.

In Table 2, the characteristics of the hollow fiber module used in this work are shown. The hollow fiber module is comprised of 10,000 microporous polypropylene fibers. The HFSLM system is shown in Fig. 2. The concentration of arsenic ions was analyzed using atomic absorption spectrometer (AAS) (model AA240FS). The pH of feed solution was measured using pH meter (model EUTECH pH 700).

**Table 2:** Characteristics of the hollow fiber module.

Characteristics	Description
Module diameter (cm)	6.3
Effective length of hollow fiber (cm)	20.3
Number of hollow fibers (–)	10,000
Inside radius of hollow fiber (cm)	0.012
Outside radius of hollow fiber (cm)	0.015
Contact area (cm^2^)	1.4 × 10^–4^
Area per unit volume (cm^−3^)	29.3
Pore size (cm)	3 × 10^–6^
Tortuosity	2.6
Porosity (%)	25
Material	Polypropylene

### Procedure

Five potential extractants: namely, Aliquat 336, TBP, DEHP, TOPO, and Cyanex 471 from the literature^2,23,38–40^ were investigated via solvent extraction. All extractants were applied at excess concentration of 0.28 M to select a suitable extractant for the extraction of arsenic ions for use in the HFSLM system. Thus, the selected extractant, mixed with toluene as the liquid membrane, was circulated along the tube and shell sides of the hollow fibers simultaneously until the hollow-fiber micropores were filled in having a total volume of about 52 mL. Then, deionized water was fed through the tube and shell sides of the hollow fibers to purge the excess liquid membrane. After that, 1 L of feed solution (synthetic produced-water) and 0.2 L of strippant solution were pumped counter-currently into the tube and shell sides of the hollow fibers, respectively. The flow pattern of feed solution was single-pass and that of strippant solution was circulating. Finally, after an interval of 10 min, both the outlet feed and strippant solutions were taken (15 mL each) to analyze the concentration of arsenic ions using AAS, which has a detection limit of 1 ppb. The deviation of analysis using AAS proved to be 0.9994. A schema of the elimination of arsenic ions via HFSLM is shown in Fig. 2.

Further, the effects of concentration of the selected extractant, pH of feed solution, types of strippant solutions, concentration of the selected strippant solution and flow rates of feed and strippant solutions were examined. After each experiment, the liquid membrane was removed from the system by feeding the surfactant into the tube and shell sides of the hollow fibers. Deionized water was then fed into the hollow fibers to eliminate the surfactant.

The efficiency of extraction and stripping were reported as percentages of extraction and stripping calculated according to Eqs. (23) and (24):

23 $$\%Extraction=\left[\frac{{\mathcal{C}}_{As,F}\left(\mathrm{0,0}\right)-{\mathcal{C}}_{As,F}\left({x}_{i},t\right)}{{\mathcal{C}}_{As,F}\left(\mathrm{0,0}\right)}\right]\times 100$$

24 $$\%Stripping=\left[\frac{{C}_{As,S}\left({\acute{x}}_{i},t\right)}{{\mathcal{C}}_{As,F}\left(\mathrm{0,0}\right)-{\mathcal{C}}_{As,F}\left({x}_{i},t\right)}\right]\times 100$$

## Results and discussion

### Effects of extractants

**Figure 3:**
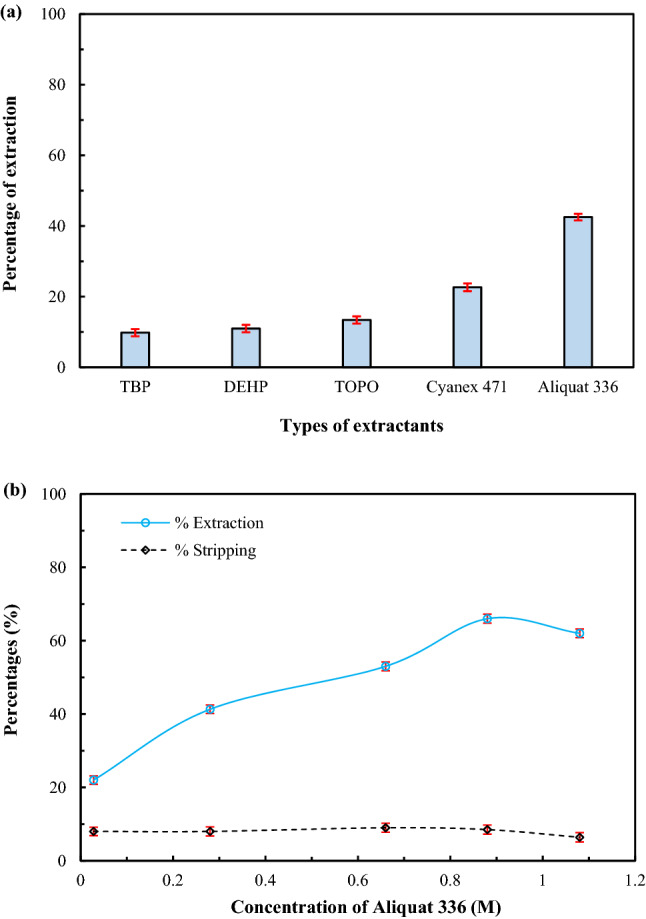
Percentages of extraction and stripping of arsenic ions using pH 6 for feed solution: (**a**) percentages of extraction against types of extractants (0.28 M each) via solvent extraction, stirring 500 rpm for 1 h; standard deviation = $\pm$ 1.0. (**b**) percentages of extraction and stripping against concentration of Aliquat 336 via HFSLM using 0.5 M NaOH as the strippant solution, at flow rates of feed and strippant solutions of 100 mL/min; standard deviations for %extraction = $\pm$ 1.18 and for %stripping = $\pm$ 1.22.

According to the literature, Aliquat 336^2^ along with the neutral extractants: TBP, DEHP, TOPO, and Cyanex 471 have been found to be potential extractants for the extraction of arsenic ions^23,38–40^. Subsequently, the extraction of arsenic ions by Aliquat 336 and neutral extractants was examined. Next, a comparison of their performance was undertaken. As shown in Fig. 3a, Aliquat 336 provided the highest extraction of arsenic ions. This result occurred due to the use of pH 6 for feed solution in the study. In such a situation, arsenic predominantly exists as dissociated species (anions)^34^. Aliquat 336 is an ionic liquid, which can extract metal ions in various species (cation, neutral and anion)^2^ but reacts much better with anions. The percentages of extraction of arsenic ions using the neutral extractants were found to be low because neutral extractants react only with undissociated species^2^. However, previous studies have found that extraction of arsenic ions using neutral extractants could be enhanced by adding H_2_SO_4_ as a co-extractant^39,40^. For studying other parameters, Aliquat 336 dissolved in toluene was used as the liquid membrane.

In Fig. 3b, the percentage of arsenic ions extraction versus concentration of Aliquat 336 is depicted. As shown, when concentration of Aliquat 336 increased, extraction of arsenic ions increased. This outcome is in accordance with Le Chatelier’s principle whereby an increase in reactant concentration results in higher fluxes. Thus, percentage of extraction reached maximum at Aliquat 336 concentration of 0.88 M. However, when concentration of Aliquat 336 increased > 0.88 M, the percentage of arsenic ions extraction decreased slightly. In general, when concentration of an extractant increases, viscosity of the liquid membrane increases, contributing to higher mass transfer resistance^41,42^. As a consequence, at some point, when the concentration of the extractant increases, the extraction does not increase.

### Effect of pH of feed solution

**Figure 4:**
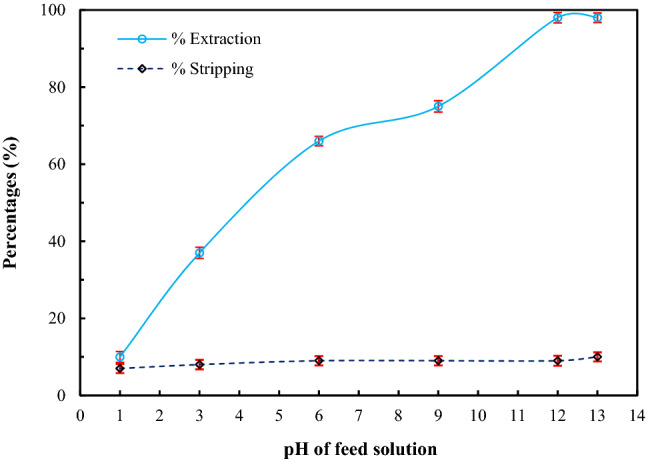
Percentages of extraction and stripping of arsenic ions against pH of feed solution via HFSLM using 0.88 M Aliquat 336 dissolved in toluene as the liquid membrane, having 0.5 M NaOH as the strippant solution, at flow rates of feed and strippant solutions of 100 mL/min; standard deviations for %extraction = $\pm$ 1.35 and for %stripping = $\pm$ 1.23.

It is acknowledged that forms of arsenic species in solution depend on the pH of the solution. As(V) predominantly exists as undissociated H_3_AsO_4_ at pH < 2 and predominantly appears as dissociated H_2_AsO_4_^–^, HAsO_4_^2–^ and ${\mathrm{AsO}}_{4}^{3-}$ at pH between 3 and 6, 8 and 11, and 12 and 14, respectively^34^. In Fig. 4, results demonstrate that extraction of arsenic ions increased as pH of feed solution increased but remained constant at pH between 12 and 13. This outcome arose due to the fact that the extraction mechanism of Aliquat 336 transpired via an ion-exchange of anion metal ions and Cl^–^ in Aliquat 336^11,43^. Undissociated H_3_AsO_4_ transforms to anions when pH of feed solution increases and predominantly appears as ${\mathrm{AsO}}_{4}^{3-}$ at pH between 12 and 13^34^, resulting in greater arsenic extraction at higher pH of feed solution. At pH 12 to 13, maximum percentage of arsenic extraction was achieved (99.9%), indicating that Aliquat 336 could extract the dissociated species ${\mathrm{AsO}}_{4}^{3-}$ much better than H_2_AsO_4_^–^ > HAsO_4_^2–^.

### Respond surface

Respond surface was applied to evaluate the optimum condition for extraction of arsenic ions using MINITAB 19 software. As shown in Table 3, the concentration of Aliquat 336 (A) and pH of feed solution (B) were coded according to 3 levels using ANOVA design. Thus, 13 runs, including 4 repetitions at the center points, were obtained.

**Table 3:** Experimental designs of the two variables: concentration of Aliquat 336 (A) and pH of feed solution (B) having 3 levels (− 1, 0 and 1); initial concentration of arsenic ions in the inlet feed solution = 4000 ppb.

Run	Level		Experimental condition		Experimental data		Data predicted by Eq. (25)	
	A	B	A	B	[As] in outlet feed	%Ex	[As] in outlet feed	%Ex
					(ppb)		(ppb)	
1	− 1	− 1	0.28	6	2320	42	2360	41
2	− 1	0	0.28	9	2120	47	2240	44
3	− 1	1	0.28	12	1320	67	1160	71
4	0	− 1	0.68	6	1880	53	1640	59
5	0	0	0.68	9	1360	66	1360	66
6	0	1	0.68	12	1	100	200	95
7	1	− 1	1.08	6	1520	62	1720	57
8	1	0	1.08	9	1520	62	1360	66
9	1	1	1.08	12	80	98	40	99
10	0	0	0.68	9	1360	66	1360	66
11	0	0	0.68	9	1360	66	1360	66
12	0	0	0.68	9	1360	66	1360	66
13	0	0	0.68	9	1360	66	1360	66

As shown in Table 3, the experimental data of arsenic ions extraction were analyzed via MINITAB to generate the regression equation for predicting the percentage of extraction of arsenic ions. The regression equation obtained from ANOVA analysis is shown in Eq. (25). ANOVA results are shown in Table 4.

25 $$\mathrm{E }= 81.1 + 95.2\mathrm{A}- 18.68\mathrm{B}- 64.9\mathrm{A}2+1.284\mathrm{B}2 + 2.29\mathrm{AB}$$

where E refers to the percentage of arsenic ions extraction, A is the concentration of Aliquat 336, and B is pH of feed solution, respectively.

**Table 4:** Analysis of variance.

Source	DF	Adj SS	Adj MS	F-value	*P* value
Model	5	3181.51	636.30	34.25	0.000
Linear	2	1635.65	817.98	44.03	0.000
A	1	0.7	0.7	0.04	0.851
B	1	1635.25	1635.25	88.02	0.000
Square	2	484.86	242.43	13.05	0.004
A^2^	1	298.13	298.13	16.05	0.005
B^2^	1	369.11	369.11	19.87	0.003
2-way interaction	1	30.25	30.25	1.63	0.243
A × B	1	30.25	30.25	1.63	0.243
Error	7	130.05	18.58		
Lack-of-fit	3	130.05	43.35		
Pure error	4	0.00	0.00		
Total	12	3311.56			

As portrayed in Table 3, the percentages of arsenic ions extraction predicted by the regression Eq. (25) were compared with those obtained from the experiment. The values predicted by Eq. (25) were found to be in accordance with the experimental results. The average relative deviation proved to be 3.77%. Therefore, the regression equation obtained via respond surface analysis can be used to predict the optimum conditions (optimum concentration of Aliquat 336 and optimum pH of feed solution) for the extraction of arsenic ions.

**Figure 5:**
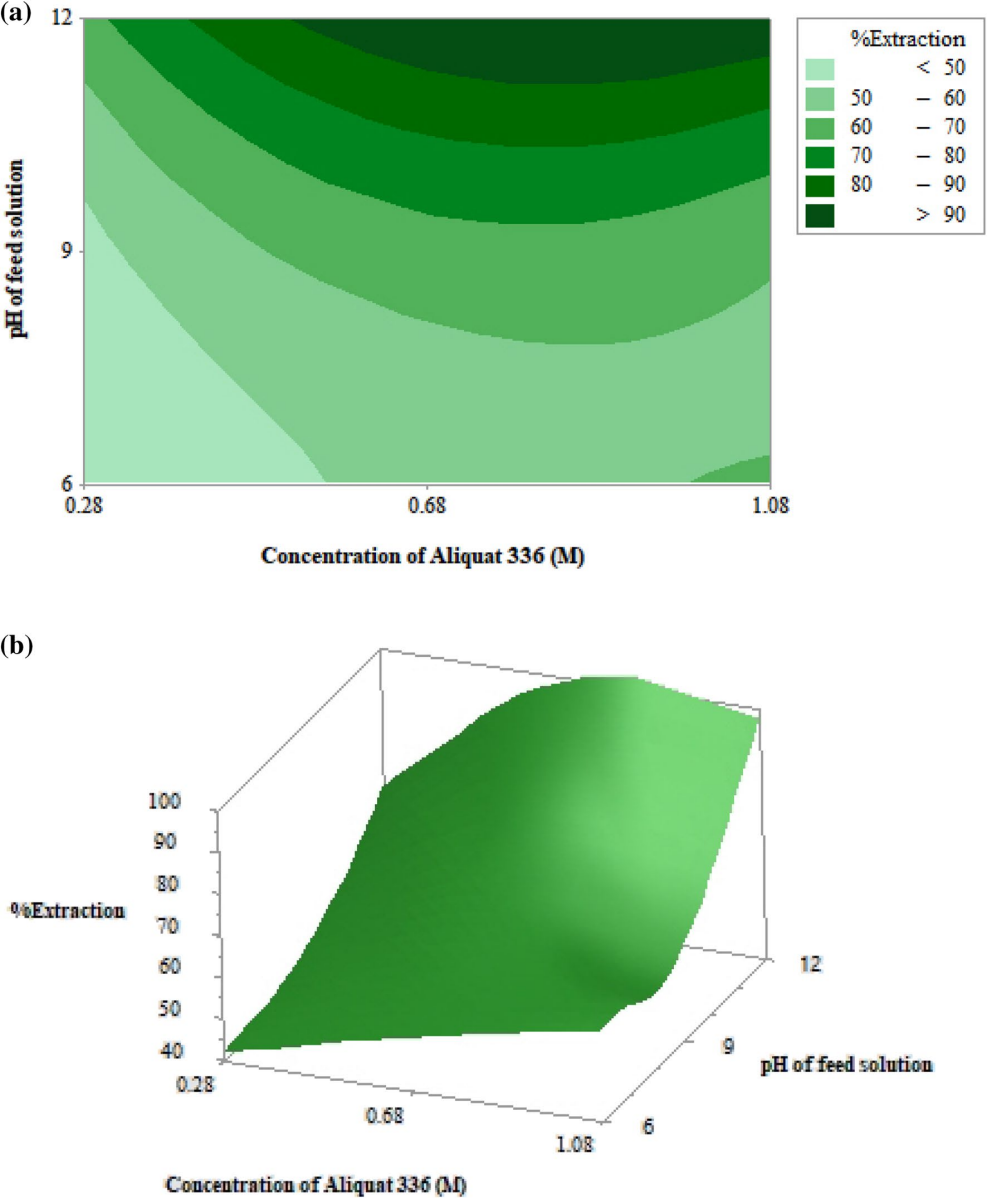
Percentages of extraction of arsenic ions against concentration of Aliquat 336 and pH of feed solution via HFSLM using 0.5 M NaOH as the strippant solution, at flow rates of feed and strippant solutions of 100 mL/min: (**a**) contour plot. (**b**) Surface plot.

In the extraction of arsenic ions, both concentration of Aliquat 336 and pH of feed solution play a vital role. In Fig. 5a,b, the contour and respond surface plots of the extraction of arsenic ions against concentration of Aliquat 336 and pH of feed solution are shown. Results demonstrate that maximum percentage of arsenic extraction (99.9%) was achieved at 0.68 M Aliquat 336 when pH of feed solution was increased from 6 to 12. Therefore, it is recommended that the pH of feed solution be adjusted to 12 and concentration of Aliquat 336 be reduced from 0.88 to 0.68 M in order to maximize the efficiency of treating arsenic and minimize the cost of chemicals. The results of this study obviously reveal that the respond surface plot is economically useful as Aliquat 336 is much more expensive than NaOH, which was used to adjust the pH of feed solution.

### Effects of strippant solutions

**Figure 6:**
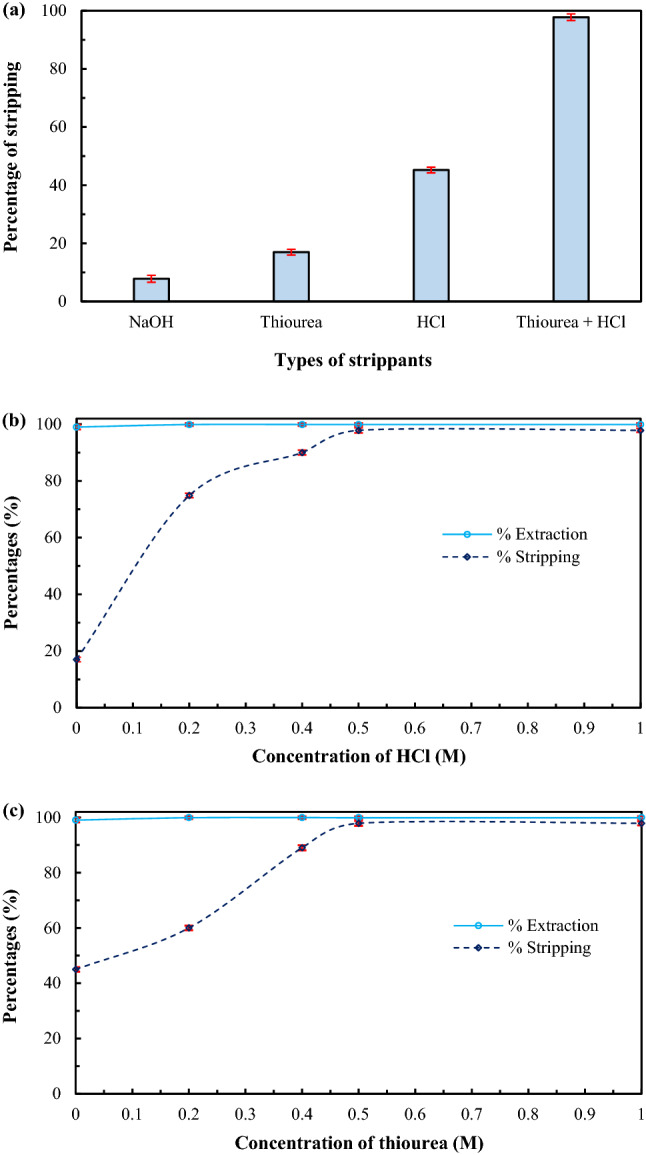
Percentages of extraction and stripping of arsenic ions using 0.68 M Aliquat 336 dissolved in toluene as the liquid membrane, having pH 12 of feed solution, at flow rates of feed and strippant solutions of 100 mL/min: (**a**) types of strippant solutions (0.5 M each); standard deviation = $\pm$ 1.07. (**b**) 0.5 M thiourea mixed with various concentrations of HCl; standard deviations for %extraction = $\pm$ 0.55 and for %stripping = $\pm$ 0.80. (**c**) 0.5 M HCl mixed with various concentrations of thiourea; standard deviations for %extraction = $\pm$ 0.55 and for %stripping = $\pm$ 0.83.

As shown in Fig. 6a, of all the strippant solutions used in this study (NaOH, thiourea and HCl), HCl was found to be the best strippant solution for the stripping of arsenic ions. Low stripping of arsenic ions using NaOH and thiourea can be explained by the fact that once arsenic ions are stripped into these strippant solutions, they exist as anions, which can then be extracted back by Aliquat 336. In the case of using HCl as strippant solution, arsenic anions, which are released into the HCl solution, combine with H^+^ resulting in H_3_AsO_4_. Such an outcome reacts poorly with Aliquat 336 in the liquid membrane phase.

When HCl was mixed with thiourea, the percentage of arsenic stripping increased from 45 to 98%. This was due to the synergistic effect^44^. To confirm the synergistic effect of stripping arsenic ions using the mixture of HCl and thiourea, the distribution ratios of arsenic stripping, using each extractant, were estimated. Results are shown in Table 5. According to Eq. (5), the synergistic coefficient was found to be 1.57, thus confirming the synergistic effect of the mixture of HCl and thiourea. The mixture of HCl and thiourea, therefore, was used as the strippant solution; their suitable concentrations will be further investigated.

**Table 5:** Distribution ratios of stripping of arsenic ions using various types of strippant solutions.

Strippant solutions	Distribution ratios
0.5 M thiourea	0.17
0.5 M HCl	0.45
0.5 M thiourea + 0.5 M HCl	0.98

In Fig. 6b,c, percentages of arsenic ions stripping against concentration of synergistic strippant solution (mixture of HCl and thiourea) are shown. Figure 6b depicts the percentage of arsenic ions stripping using 0.5 M thiourea having various concentrations of HCl. As concentration of HCl increased, the percentage of stripping of arsenic ions increased corresponding to chemical kinetics. At 0.5 M HCl, the percentage of stripping of arsenic ions reached maximum at 98%, and remained constant thereafter. Figure 6c depicts the percentage of stripping of arsenic ions using 0.5 M HCl having various concentrations of thiourea. Consequently, as thiourea concentration increased, the percentage of stripping of arsenic ions increased, in accordance with chemical kinetics. When concentration of thiourea was higher than 0.5 M, the percentage of stripping also remained constant at 98%. Therefore, 0.5 M HCl and 0.5 M thiourea were used in the study.

### Reaction order and reaction rate constant

Integration and graphical methods, plotted between the integral concentration of arsenic ions versus time, were utilized to estimate reaction rate constants and reaction orders of arsenic extraction and stripping. Results are given in Table 6. Reactions, therefore, for both extraction and stripping of arsenic ions from synthetic produced water were found to be of second-order: due to the highest R^2^.

**Table 6:** Reaction order (*m*/*n*) and reaction rate constants of extraction and stripping of arsenic ions (*k*_*As,Ex*_ and *k*_*As,S*_).

*m/n*	Reaction	Plot	Rate constant	R^2^
1	Extraction	$ln\frac{{C}_{As,0}}{{C}_{As}}$ versus *t*	28.41 × 10^–3^ s^−1^	0.8957
	Stripping	$ln\frac{{C}_{As,Org}}{{{C}_{As,Org}-C}_{As,S}}$ versus *t*	9.90 × 10^–3^ s^−1^	0.9669
2	Extraction	$\frac{1}{{C}_{As}}-\frac{1}{{C}_{As,0}}$ and *t*	50.64 × 10^–3^ L/mg s	0.9934
	Stripping	$\frac{1}{{{C}_{As,Org}-C}_{As,S}}-\frac{1}{{C}_{As,Org}}$ versus *t*	6.96 × 10^–3^ L/mg s	0.9952
3	Extraction	$\frac{1}{{2C}_{As}^{2}}-\frac{1}{2{C}_{As,0}^{2}}$ and *t*	16.67 × 10^–2^ L^2^/mg^2^ s	0.9893
	Stripping	$\frac{1}{2{\left({{C}_{As,Org}-C}_{As},S\right)}^{2}}-\frac{1}{2{C}_{As,Org}^{2}}$ versus *t*	5.52 × 10^–2^ L^2^/mg^2^ s	0.9895

*C*_*As*_, concentration of arsenic ions in the feed solution at time *t*; *C*_*As,0*_, concentration of arsenic ions in the inlet feed solution; *C*_*As,Org*_, initial concentration of arsenic ions in the organic extractant; *C*_*As,S*_, concentration of arsenic ions in the stripping solution at time *t.*

**Figure 7:**
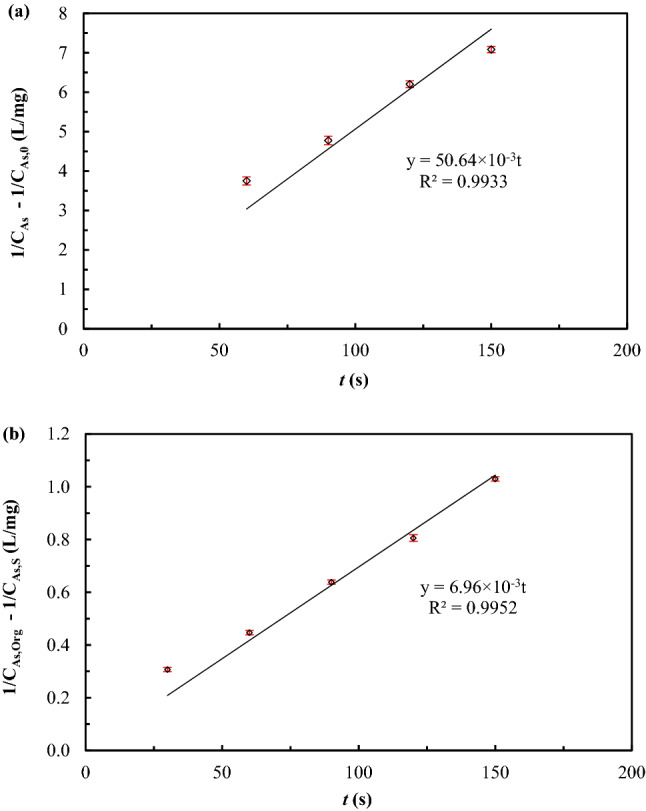
Graphs plotted between the integral concentration of arsenic ions versus time: (**a**) extraction reaction; standard deviations = $\pm$ 0.09. (**b**) Stripping reaction standard deviations = $\pm$ 0.009.

As shown in Fig. 7, according to the plots between the integral concentration of arsenic versus time, the reaction rates of extraction and stripping of arsenic ions were 50.64 × 10^–3^ and 6.96 × 10^–3^ L/mg.s, respectively. These values indicate that the rate of stripping is slower that the rate of extraction.

### Validation of the mathematical model

**Figure 8:**
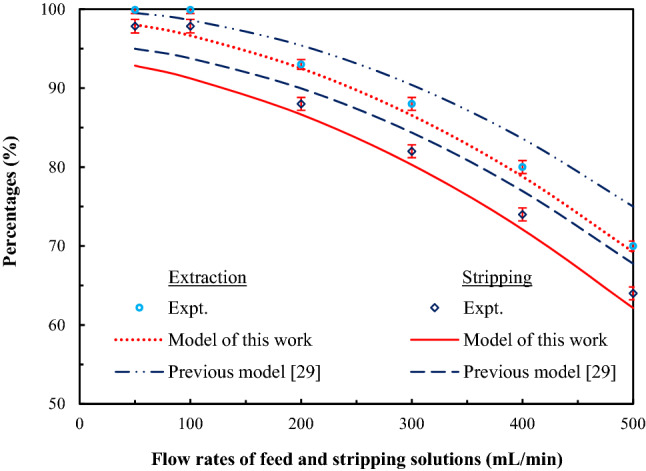
Percentages of extraction and stripping of arsenic ions against flow rates of feed and strippant solutions via HFSLM having 0.68 M Aliquat 336 dissolved in toluene as the liquid membrane, at pH 12 of feed solution, using a mixture of HCl and thiourea (0.5 M each) as the strippant solution; standard deviations for %extraction = $\pm$ 0.6 and for %stripping = $\pm$ 0.8.

Various flow rates of feed and strippant solutions between 50 and 500 mL/min were investigated in order to optimize the elimination of arsenic ions and validate the mathematical model developed in this work. As shown in Fig. 8, at flow rates between 50 and 100 mL/min, percentages of extraction and stripping of arsenic ions remained constant at 99.9% and 98%, respectively. The concentration of arsenic ions in the outlet feed solution was found to be 1 ppb. Notably, this result complied with the standard of wastewater discharged, as regulated by the Ministry of Industry and the Ministry of Natural Resources and Environment, Thailand. Subsequently, due to less residence time^45^, percentages of extraction and stripping declined when flow rates increased. Therefore, flow rates of feed and strippant solutions of 100 mL/min were recommended in order to optimize the efficiency and capacity of arsenic ions treatment.

In Fig. 8, percentages of extraction and stripping of arsenic ions obtained from the model and the experiment are also compared. Results clearly show that the mathematical model developed in this work agreed well with the experimental results having average relative deviations of 2% and 4% for predicting the extraction and stripping, respectively. These values confirm that chemical reactions at the liquid–membrane interfaces as well as diffusion are significant factors governing the rate of arsenic ions transport across the liquid membrane.

Moreover, percentages of extraction and stripping of arsenic ions obtained from the model developed in this work were compared with those obtained from the model developed previously^29^. As results show in Fig. 8, the mathematical model developed herein proved to be in agreement with the experimental results more than the previous model^29^. This outcome arose because the previous model ignored the significant factor (diffusion). Thus, both diffusion and chemical reactions were found to be key factors controlling the transport rate of arsenic ions across the hollow fibers.

### Adaptability of the mathematical model

**Figure 9:**
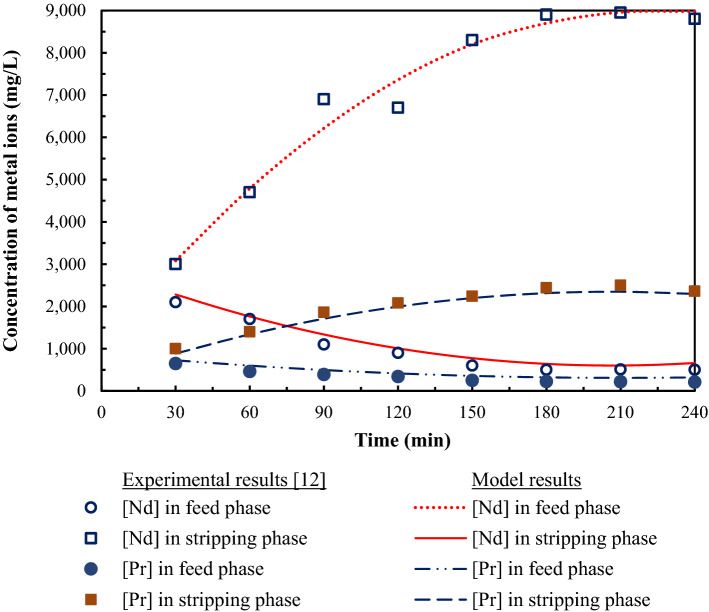
Adaptability of the mathematical model to predict the extraction and stripping of other metal ions via HFSLM.

To ensure the mathematical model developed in this work can be adapted for use with other metal ions, the model was utilized to predict the extraction and stripping of neodymium (Nd) and praseodymium (Pr) using HFSLM. As results show in Fig. 9, the concentration of Nd and Pr, in both feed and strippant phases, predicted by the model developed in this work was in good agreement with the experimental results carried out by Ni’am et al.^12^. The results, as shown in Figs. 8 and 9, indicate that the mathematical model, herein, can predict the extraction and stripping of various metal ions via HFSLM.

## Conclusion

In this paper, results demonstrated that HFSLM could successfully treat arsenic ions in synthetic wastewater to meet the standard of wastewater discharged (< 250 ppb). Arsenic ions were extracted and stripped in a single-step operation. Applying optimum conditions, percentages of extraction and stripping of arsenic ions reached 99.9% and 98%, respectively. Besides, extraction and stripping of arsenic ions predicted by the mathematical model fitted in well with the experimental results. The average relative deviations for predicting the extraction and stripping proved to be 2% and 4%, respectively. This confirms that chemical reactions at the liquid–membrane interfaces and diffusion are the crucial parameters that govern the rate of arsenic ions transport across the liquid membrane.

## Supplementary Information

Supplementary Information.
