# Supplementary material for: The elimination of trace arsenic via hollow fiber supported liquid membrane: experiment and mathematical model
Source: Sci Rep. 2021 Jun 3;11:11790. doi: 10.1038/s41598-021-91326-9 (PMC8175572; doi:10.1038/s41598-021-91326-9)
Supplement: Supplementary file 1 — Supplementary Information. [file 41598_2021_91326_MOESM1_ESM.pdf]

The Elimination of Trace Arsenic via Hollow Fiber Supported Liquid Membrane: Experiment and Mathematical Model

Sira Suren, Watcharapong Ampronpong, Ura Pancharoen and Kreangkrai Maneeintr

Appendix A.

Mathematical details: transport of arsenic ions across HFSLM

In the feed phase, the conservation of mass of arsenic ions in each tiny segment (Δx) is defined as:

$$q_F C_{As,F}(x_{i-1},t) - q_F C_{As,F}(x_i,t) + \frac{A_{c,F}\mathfrak{D}_{As,F}}{\Delta x}\Big(C_{As,F}(x_i,t) - C_{As,F}(x_{i-1},t)\Big) + r_{As,Ex}(x_i,t)V_F = V_F \frac{dC_{As,F}(x_i,t)}{dt} \quad \text{(A.1)}$$

where

$$A_{c,F} = \pi r_i^2$$

$$V_F = \pi r_i^2 \Delta x$$

$$\Delta x = \frac{L}{i}$$

r_i and L refer to the inside radius and effective length of the hollow fibers, respectively.

The reaction rate of arsenic extraction viz $r_{As,Ex}(x_i,t)$, shown in Eq. (A.1), can be expressed as follows:

$$r_{As,Ex}(x_i,t) = -k_{Ex}C_{As,F}^{m}(x_i,t) \qquad \text{(A.2)}$$

By linearizing $r_{As,Ex}(x_i,t)$ in Eq. (A.2), using the Taylor series, the following equation is obtained:

$$r_{As,Ex}(x_i,t) = -\left[\Omega C_{As,F}(x_i,t) + \psi\right] \qquad \text{(A.3)}$$

where

$\Omega = mk_{Ex}C_{As,F}^{m-1}(0,0)$

$\psi = (1-m)k_{Ex}C_{As,F}^{m}(0,0)$

$C_{As,F}(0,0)$ stands for the concentration of arsenic ions in the inlet feed solution.

Substituting Eq. (A.3) into (A.1) and then rearranging the equation yields:

$$\frac{V_F}{q_F}\frac{dC_{As,F}(x_i,t)}{dt} = \left(1 - \frac{A_{c,F}\mathfrak{D}_{As,F}}{\Delta x}\right)C_{As,F}(x_{i-1},t) - \left(1 + \frac{V_F\Omega}{q_F} - \frac{A_{c,F}\mathfrak{D}_{As,F}}{\Delta x}\right)C_{As,F}(x_i,t) - \frac{V_F\psi}{q_F} \qquad \text{(A.4)}$$

The conservation of mass of arsenic ions in tiny segments 1, 2, 3, …, *i* based on Eq (A.4) is as follows:

$$\frac{V_F}{q_F}\frac{dC_{As,F}(x_1,t)}{dt} = \left(1 - \frac{A_{c,F}\mathfrak{D}_{As,F}}{\Delta x}\right) C_{As,F}(x_0,t) - \left(1 + \frac{V_F\Omega}{q_F} - \frac{A_{c,F}\mathfrak{D}_{As,F}}{\Delta x}\right) C_{As,F}(x_1,t) - \frac{V_F\psi}{q_F} \quad (A.5)$$

$$\frac{V_F}{q_F}\frac{dC_{As,F}(x_2,t)}{dt} = \left(1 - \frac{A_{c,F}\mathfrak{D}_{As,F}}{\Delta x}\right) C_{As,F}(x_1,t) - \left(1 + \frac{V_F\Omega}{q_F} - \frac{A_{c,F}\mathfrak{D}_{As,F}}{\Delta x}\right) C_{As,F}(x_2,t) - \frac{V_F\psi}{q_F} \quad (A.6)$$

$$\frac{V_F}{q_F}\frac{dC_{As,F}(x_3,t)}{dt} = \left(1 - \frac{A_{c,F}\mathfrak{D}_{As,F}}{\Delta x}\right) C_{As,F}(x_3,t) - \left(1 + \frac{V_F\Omega}{q_F} - \frac{A_{c,F}\mathfrak{D}_{As,F}}{\Delta x}\right) C_{As,F}(x_3,t) - \frac{V_F\psi}{q_F} \quad (A.7)$$

.

.

.

$$\frac{V_F}{q_F}\frac{dC_{As,F}(x_i,t)}{dt} = \left(1 - \frac{A_{c,F}\mathfrak{D}_{As,F}}{\Delta x}\right) C_{As,F}(x_{i-1},t) - \left(1 + \frac{V_F\Omega}{q_F} - \frac{A_{c,F}\mathfrak{D}_{As,F}}{\Delta x}\right) C_{As,F}(x_i,t) - \frac{V_F\psi}{q_F} \quad (A.8)$$

The series of differential equations i.e. (A.5) to (A.8) can be solved using the concept of the Generating Function, which can be defined as in Eq. (A.9):

$$\mathcal{C}_{As}(z,t) = \mathcal{C}_{As,0}Z^0 + \mathcal{C}_{As,1}Z^1 + \mathcal{C}_{As,2}Z^2 + \cdots \quad (A.9)$$

where

$$\mathcal{C}_{As}(z,0) = \mathcal{C}_{As}(x_0,0)Z^0 + \mathcal{C}_{As}(x_1,0)Z^1 + \mathcal{C}_{As}(x_2,0)Z^2 + \cdots$$

$$= \mathcal{C}_{As}(x_0,0)Z^0 = \mathcal{C}_{As}(x_0,0) = \mathcal{C}_{As}(0,0)$$

Z is a complex quantity.

Equation (A.9) can be represented as:

$$\frac{\partial \mathcal{C}_{As}(z,t)}{\partial t} = Z^0\frac{d\mathcal{C}_{As}(x_0,t)}{dt} + Z^1\frac{d\mathcal{C}_{As}(x_1,t)}{dt} + Z^2\frac{d\mathcal{C}_{As}(x_2,t)}{dt} + Z^3\frac{d\mathcal{C}_{As}(x_3,t)}{dt} + \cdots \quad (A.10)$$

Multiplying Eqs. (A.5) to (A.8) by Z^1, Z^2, Z^3, …, Z^i and then summarizing them gives:

$$\frac{V_F}{q_F}\left[\frac{dC_{As,F}(x_0,t)}{dt}+Z\frac{dC_{As,F}(x_1,t)}{dt}+Z^2\frac{dC_{As,F}(x_2,t)}{dt}+Z^3\frac{dC_{As,F}(x_3,t)}{dt}+\cdots\right]=$$

$$Z[\omega C_{As}(x_0,t)+Z\omega C_{As}(x_1,t)+Z^2\omega C_{As}(x_2,t)+\cdots]\ -$$

$$\vartheta[C_{As}(x_0,t)+ZC_{As}(x_1,t)+Z^2C_{As}(x_2,t)+Z^3C_{As}(x_3,t)+\cdots]\ -$$

$$\frac{V_F}{q_F}\psi[Z+Z^2+Z^3+\cdots]+\vartheta C_A(x_0,t) \qquad \text{(A.11)}$$

where

$$\omega=1-\frac{A_{c,F}\mathfrak{D}_{As,F}}{\Delta\ x}$$

$$\vartheta=1+\frac{V_F\Omega}{q_F}-\frac{A_{c,F}\mathfrak{D}_{As,F}}{\Delta x}$$

As per the definition of the Generating Function, Eq. (A.11) can be represented as:

$$\frac{V_F}{q_F}\frac{\partial\mathcal{C}_{As,F}(z,t)}{\partial t}=Z\omega\mathcal{C}_{As,F}(Z,t)-\vartheta\mathcal{C}_{As,F}(Z,t)-\frac{V_F}{q_F}\psi\sum_{j=1}^{i}Z^j+\vartheta\mathcal{C}_{As,F}(x_0,t) \qquad \text{(A.12)}$$

Integrating Eq. (A.12) using the boundary condition in Eq. (A.9) obtains:

$$\mathcal{C}_{As,F}(z,t)=\left[\frac{Z\omega\mathcal{C}_{As,F}(0,0)e^{Z\omega\frac{tq_F}{V_F}}}{(Z\omega-\vartheta)}-\frac{V_F\psi\sum_{j=1}^{i}Z^je^{Z\omega\frac{tq_F}{V_F}}}{q_F(Z\omega-\vartheta)}\right]e^{-\vartheta\frac{tq_F}{V_F}}+\frac{V_F\psi\sum_{j=1}^{i}Z^j}{q_F(Z\omega-\vartheta)}-\frac{\vartheta\mathcal{C}_{As,F}(0,0)}{(Z\omega-\vartheta)} \qquad \text{(A.13)}$$

By distributing the Z function in Eq. (A.13) into polynomial form and using the method of undetermined coefficients, an equation for estimating the concentration of arsenic ions in the outlet feed solution, $C_{As,F}(x_i, t)$, is obtained, as shown in Eq. (A.14):

$$C_{As,F}(x_i,t) = -C_{As,F}(0,0)e^{-\vartheta\frac{tq_F}{V_F}}\sum_{k=1}^{i}\frac{1}{(i-k)!}\left(\frac{1}{\vartheta}\right)^{k}\left(\frac{tq_F}{V_F}\right)^{i-k}\omega^{i} + \frac{V_F\psi e^{-\vartheta\frac{tq_F}{V_F}}}{q_F\vartheta}\sum_{k=1}^{i}\frac{1}{(i-k)!}\left(\frac{tq_F\omega}{V_F}\right)^{i-k}\sum_{l=1}^{k}\left(\frac{\omega}{\vartheta}\right)^{l-1} - \frac{V_F\psi\sum_{k=1}^{i}\omega^{i-k}}{q_F\vartheta^{i-k+1}} + C_{As,F}(0,0)\left(\frac{\omega}{\vartheta}\right)^{i} \quad \text{(A.14)}$$

For the strippant phase, the mass conservation of arsenic ions in each tiny segment ($\Delta\acute{x}$) is defined as:

$$q_S C_{As,S}(\acute{x}_{i-1},t) - q_S C_{As,S}(\acute{x}_i,t) + \frac{A_{c,S}\mathfrak{D}_{As,S}}{\Delta\acute{x}_i}\left(C_{As,S}(\acute{x}_i,t) - C_{As,S}(\acute{x}_{i-1},t)\right) + r_{As,S}(\acute{x}_i,t)V_S = V_S\frac{dC_{As,S}(\acute{x}_i,t)}{dt} \quad \text{(A.15)}$$

where

$$A_{c,S} = \frac{\sqrt{3}}{4}d_0^2 - \frac{\pi r_0^2}{2}$$

$$V_S = A_{c,S}\Delta\acute{x}$$

$$\Delta\acute{x} = \Delta x = \frac{L}{i}$$

r_o is the outside radius and d_o is the outside diameter of the hollow fibers.

The reaction rate of arsenic ions stripping, $r_{As,S}(x_i,t)$, in Eq. (A.15), which is defined as in Eq. (4), can be linearized using the Taylor series. The linearized equation is shown below:

$$r_{As,S}(\acute{x}_i,t) = \sigma C_{As,S}(\acute{x}_i,t) + \delta \tag{A.16}$$

where $\sigma = nk_S C_{As,S}^{n-1}(0,0)$, and $\delta = (1-n)k_S C_{As,S}^{m}(0,0)$

Merging Eqs. (A.16) and (A.15) and rearranging the equation achieves:

$$\frac{V_S}{q_S}\frac{dC_{As,S}(\acute{x}_i,t)}{dt} = \left(1 - \frac{A_{c,S}\mathfrak{D}_{As,S}}{\Delta\acute{x}_i}\right) C_{As,S}(\acute{x}_{i-1},t) - \left(1 - \frac{V_S\sigma}{q_S} - \frac{A_{c,S}\mathfrak{D}_{As,S}}{\Delta\acute{x}_i}\right) C_{As,S}(\acute{x}_i,t) + \frac{V_S\delta}{q_S} \tag{A.17}$$

The conservation of mass of arsenic ions in tiny segments 1, 2, 3, …, *i* based on Eq. (A.17) is as follows:

$$\frac{V_S}{q_S}\frac{dC_{As,S}(\acute{x}_1,t)}{dt} = \left(1 - \frac{A_{c,S}\mathfrak{D}_{As,S}}{\Delta x}\right) C_{As,S}(\acute{x}_0,t) - \left(1 - \frac{V_S\sigma}{q_S} - \frac{A_{c,S}\mathfrak{D}_{As,S}}{\Delta\acute{x}}\right) C_{As,S}(\acute{x}_1,t) + \frac{V_S\delta}{q_S} \tag{A.18}$$

$$\frac{V_S}{q_S}\frac{dC_{As,S}(\acute{x}_2,t)}{dt} = \left(1 - \frac{A_{c,S}\mathfrak{D}_{As,S}}{\Delta x}\right) C_{As,S}(\acute{x}_1,t) - \left(1 - \frac{V_S\sigma}{q_S} - \frac{A_{c,S}\mathfrak{D}_{As,S}}{\Delta\acute{x}}\right) C_{As,S}(\acute{x}_2,t) + \frac{V_S\delta}{q_S} \tag{A.19}$$

$$\frac{V_S}{q_S}\frac{dC_{As,S}(\acute{x}_3,t)}{dt} = \left(1 - \frac{A_{c,S}\mathfrak{D}_{As,S}}{\Delta x}\right) C_{As,S}(\acute{x}_2,t) - \left(1 - \frac{V_S\sigma}{q_S} - \frac{A_{c,S}\mathfrak{D}_{As,S}}{\Delta\acute{x}}\right) C_{As,S}(\acute{x}_3,t) + \frac{V_S\delta}{q_S} \tag{A.20}$$

.

.

.

$$\frac{V_S}{q_S}\frac{dC_{As,S}(\acute{x}_i,t)}{dt} = \left(1 - \frac{A_{c,S}\mathfrak{D}_{As,S}}{\Delta\acute{x}}\right) C_{As,S}(\acute{x}_{i-1},t) - \left(1 - \frac{V_S\sigma}{q_S} - \frac{A_{c,S}\mathfrak{D}_{As,S}}{\Delta\acute{x}}\right) C_{As,S}(\acute{x}_i,t) + \frac{V_S\delta}{q_S} \tag{A.21}$$

Solving the series of differential equations in Eqs. (A.18) to (A.21), using the same concept as described in Eqs. (A.5) to (A.14), yields the equation for determining the concentration of arsenic ions in the outlet strippant solution ($C_{As,S}(\acute{x}_i, t)$), as shown below:

$$C_{As,S}(\acute{x}_i, t) = -C_{As,S}(0,0)e^{-\zeta\frac{tQ_S}{V_S}} \sum_{k=1}^{i} \frac{1}{(i-k)!}\left(\frac{1}{\zeta}\right)^{k} \cdot \left(\frac{tQ_S}{V_S}\right)^{i-k} \varpi^{i} -$$

$$\frac{\delta V_S e^{-\zeta\frac{V_S}{Q_S}}}{Q_S\lambda} \sum_{k=1}^{i} \frac{1}{(i-k)}\left(\frac{tQ_S\varpi}{V_S}\right)^{i-k} \sum_{l=1}^{k}\left(\frac{\varpi}{\zeta}\right)^{l-1} + \frac{\delta V_S}{Q_S}\sum_{k=1}^{i}\frac{\varpi^{i-k}}{\zeta^{i-k+1}} + C_{As,S}(0,0)\left(\frac{\varpi}{\zeta}\right)^{i} \quad \text{(A.22)}$$

where

$$\varpi = 1 - \frac{A_{c,S}\mathfrak{D}_{As,S}}{\Delta\acute{x}}$$

$$\zeta = \left(1 - \frac{V_S\sigma}{q_S} - \frac{A_{c,S}\mathfrak{D}_{As,S}}{\Delta\acute{x}}\right)$$

$C_{As,S}(0,0)$ is the concentration of arsenic ions in the strippant phase at time zero, which can be calculated by Eq. (A.23).

$$C_{As,S}(0,0) = \frac{(k_m + k_{Ex})C_{As,F}(0,0) - (k_m + 2k_{Ex})C_{As,F}(x_i,t)}{k_m} \quad \text{(A.23)}$$

where k_m is the liquid-membrane mass-transfer coefficient, which can be estimated as follows:

$$k_m = \frac{\varepsilon\mathfrak{D}_{As}}{r_i\tau ln(r_0/r_i)} \quad \text{(A.24)}$$

where ε and τ refers to porosity and tortuosity of the hollow fibers, respectively.
